# Sequence Complexity of Chromosome 3 in *Caenorhabditis elegans*

**DOI:** 10.1155/2012/287486

**Published:** 2012-07-20

**Authors:** Gaetano Pierro

**Affiliations:** System Biology, PhD School, University of Salerno, Via Ponte Don Melillo, 84084 Fisciano, Italy

## Abstract

The nucleotide sequences complexity in chromosome 3 of *Caenorhabditis elegans* (*C. elegans*) is studied. The complexity of these sequences is compared with some random sequences. Moreover, by using some parameters related to complexity such as fractal dimension and frequency, indicator matrix is given a first classification of sequences of *C. elegans*. In particular, the sequences with highest and lowest fractal value are singled out. It is shown that the intrinsic nature of the low fractal dimension sequences has many common features with the random sequences.

## 1. Introduction

The *Caenorhabditis elegans* (*C*. *elegans*) is a 1 mm length transparent nematode. Thanks to its simple organic structure, it was taken as a model for research into genetic field. Early studies on *C*. *elegans* began in 1962 with some works on cell lineage and apoptosis [1, 2]. There are 2 distinct sexual types of the *C*. *elegans*, the hermaphrodite and the male. The second one is very rarely represented in nature (being approximately only the 0.05% of the population). We have 959 cells in the hermaphroditic species and 1031 cells for the male. The sexual difference at the chromosomal level provides: XX chromosomes for hermafrodite and X0 for the male. The sexual reproduction of *C*. *elegans* is realized by 2 distinct pathways: mating or, in case of the hermaphrodite, by a self-fertilization. The life cycle of *C*. *elegans* consists of 4 larval stages (from L1 to L4); however, if there exists some hard environment conditions, such as lacking of food, the *C*. *elegans* remains in the L3 larval stage, until the conditions improve.

The complete sequencing of *C*. *elegans* genome was completed in 2002. The *C*. *elegans* has 5 chromosomes autosomes plus the sex chromosome X. Totally, it is made up of nearly 100 million base pairs and 19000 genes [3–5]. Study on fractal analysis of multigenome of *C*. *elegans* has shown that chromosome 3 is the one with multifractal characteristics higher than the others, the less multifractal appears to be the chromosome sexual X [6]. For the first time, in this work, we have analyzed the different types of sequences belonging to the genome of *C*. *elegans*, focusing our investigation on those that show fractal characteristics. Thus, chromosome 3 of *C*. *elegans* has been carefully studied because its unsymmetrical and inhomogeneous statistical characteristics. Through the analysis of this chromosome we can investigate what are the features that make it more “complex” from a biostatistical point of view and in particular with the use of statistical parameters such as the complexity, the fractal dimension, the matrix correlation, and the nucleotide frequency. The concept of fractality in biology is further clarified.

On the chromosome 3 of *C*. *elegans,* 2780 genes have been identified. In this paper, almost all nucleotide sequences that are located on chromosome 3 of *C*. *elegans* were analyzed and compared with random sequences. In particular, it will be shown that the nucleotide sequences with a low fractal value have common features with random sequence with low fractal dimension. Moreover, the highest fractal dimension corresponds to sequence close to random sequence with high fractal value, and in particular, it is shown a high frequency of cytosine.

From mathematical point of view, a fractal is a geometric object, characterized by the self-similarity; that is, it repeats its structure cyclically in the same way at different scales. A more rigorous definition of a fractal is based on four properties: self-similarity, fine structure, irregularities, and noninteger dimension [7]. The fractal dimension is a parameter to compute the degree of complexity or disorder by measuring the unsmoothness of the object. This value enables to measure the amount of information contained in the sequence, the higher value corresponds to a higher information content. Generally, this value ranges between 1 and 2, so that the higher value corresponds to the higher complexity. Fractality has been observed and measured in pathology and cancer models [8, 9], the study of branching blood vessels, or the irregularity of the contours of tumor cells [10, 11], the analysis of complete genomes [12], the correlation analysis of protein sequences [13] tissue pathology [14], in exons, introns [15], and nuclei [16], and it is involved in blood cancer [17, 18].

## 2. Materials and Methods

In the chromosome 3 of *C*. *elegans,* there have been singled out 2780 genes [19]. Some of them are very short, less than about 50 nucleotides, thus being useless for any statistical analysis, and some of them are still under investigation, so that some nucleotides are not yet properly identified. For this reason, there have been selected only some sequences with significant length, the shortest being about 100 nucleotides. In particular, we investigated 100 genes (whole sequence), 85 repeats sequences, 71 noncoding sequences (introns), and 100 coding sequences (exons lacks of UTR). In order to make a comparison with random sequences, 100 random sequences of 100 nucleotides have been generated. In this work, all sequences were downloaded from the National Center for Biotechnology Information [19]. A simple formula to estimate the fractal dimension has been given in [20, 21] and based on the correlation matrix, as follows. The fractal dimension is defined as the average of the number *p*(*n*) of 1 in the randomly taken *n* × *n* minors of the *N* × *N* correlation matrix *u*_*hk*_ (see also [20–24]).

In particular, let

(1) $$\begin{array}{c} {ℵ}_{4}=\left\{A,C,G,T\right\} \end{array}$$

be the finite set (alphabet) of nucleotides and *x* ∈ *ℵ*_4_ any member of the 4 symbols alphabet.

A DNA sequence is the finite symbolic sequence *𝔇*(*N*) = *¥* × *ℵ*_4_ so that

(2) $$\begin{array}{c} 𝔇\left(N\right)={\left\{x_{h}\right\}}_{h=1,\ldots ,N},\;N<\infty \end{array}$$

being

(3) $$\begin{array}{c} x_{h}=\left(h,x\right)=x\left(h\right),\;\left(h=1,2,\ldots ,N;\;x\in {ℵ}_{4}\right) \end{array}$$

the acid nucleic *x* at the position *h*.

Let *𝔇*_1_(*N*), *𝔇*_2_(*N*) be two DNA sequences, the indicator function [20, 22–26] is the map

(4) $$\begin{array}{c} u:{𝔇}_{1}\left(N\right)\times {𝔇}_{2}\left(N\right)\to \left\{0,1\right\} \end{array}$$

such that the correlation matrix

(5) uhk=u(xh,xk)={1,if  xh=xk,0,if  xh≠xk,(xh∈𝔇1(N), xk∈𝔇2(N))

is a matrix of 0's and 1's showing the existence of correlation. When *𝔇*_1_(*N*) ≡ *𝔇*_2_(*N*), the indicator function shows the existence of autocorrelation on the same sequence.

The probability distribution of nucleotides can be defined by the frequency

(6) $$\begin{array}{c} p_{X}\left(n\right)=\frac{1}{n}\sum_{i=1}^{n}u_{Xi},\;\left(X\in {ℵ}_{4},\;x_{i}\in 𝔇\left(N\right);\;1\leq n\leq N\right) \end{array}$$

that the acid nucleic *X* can be found at the position *n*. This value can be approximated by the frequency count (on the indicator matrix) of the nucleotide distribution before *n* [20, 21, 23, 24]

(7) $$\begin{array}{c} D=\frac{1}{2}\frac{1}{N}\sum_{n=2}^{N}\frac{\log p\left(n\right)}{\log n}. \end{array}$$

In order to have a measure of complexity, for an *n*-length sequence, we use the following definition [20–24]:

(8) $$\begin{array}{c} K=\log{\left(\frac{n!}{a_{n}!c_{n}!g_{n}!t_{n}!}\right)}^{1/n} \end{array}$$

with

(9) an=∑h=1,…,nu(A,xh),  cn=∑h=1,…,nu(C,xh),gn=∑h=1,…,nu(G,xh),  tn=∑h=1,…,nu(T,xh).

## 3. Results

By using formula (7), for each sequence of nucleotides, the corresponding fractal dimension has been computed, and obtained results are shown in Tables 1 and 2. In particular, the sequences with max/min values of fractal dimension among the whole sequences, coding/noncoding sequences, repeat sequences, random sequences have been singled out.

**Figure 1:**
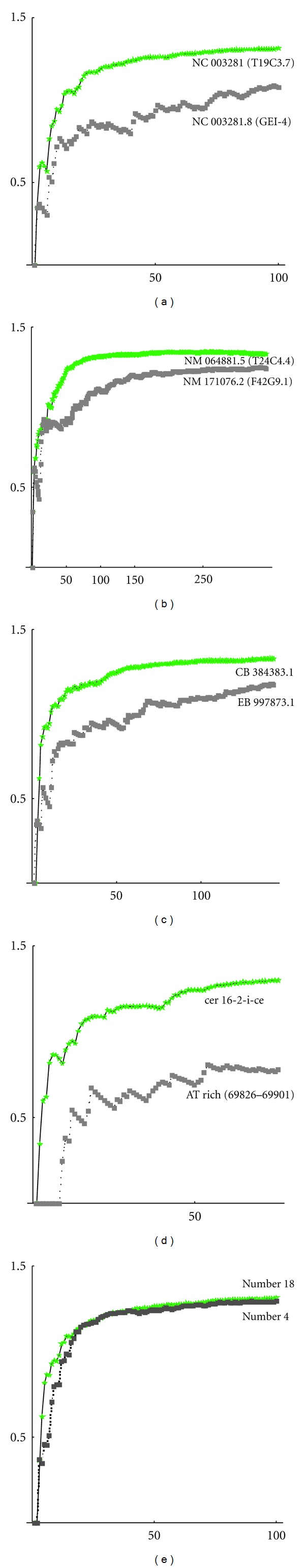
Curves of min-max complexity: (a) whole gene, (b) noncoding, (c) coding, (d) repeats, and (e) random sequences.

From these computations, we can see that the repeats sequence AT rich (69826–69901) has the lowest fractal value 1.24155. This could be explained because we have a large number of only 2 nucleotides, so that the sequence is simple in the sense that there is a low variability and it shows a low complexity. Analogously, the sequence with the highest value of fractality is still a repeats sequence CER 16-2-i-CE with a fractal dimension 1.31280. Although there are some fluctuations, due to the fact that random generation, by a computer, is indeed a pseudorandom generation, the values of fractal dimension for random sequences are localized around 1.28, which appears to be the intermediate value between the maximum and minimum values obtained for all sequences examined. Further information about the heterogeneity of data is given by the complexity parameter (8). In Figure 1, the complexity curves corresponding to the sequences for maximum and minimum values of the fractal are plotted.

**Figure 2:**
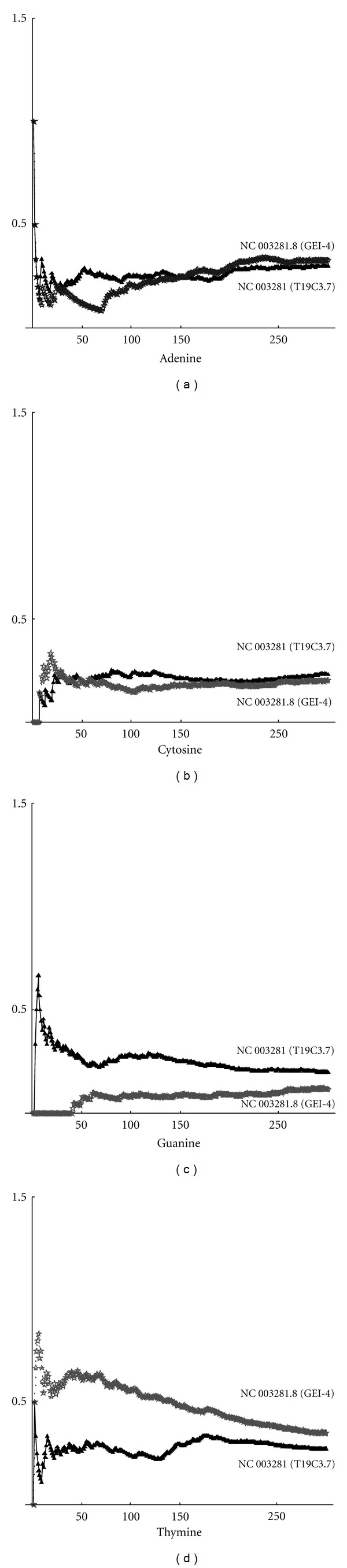
Max-min frequency curves for the whole sequence.

**Figure 3:**
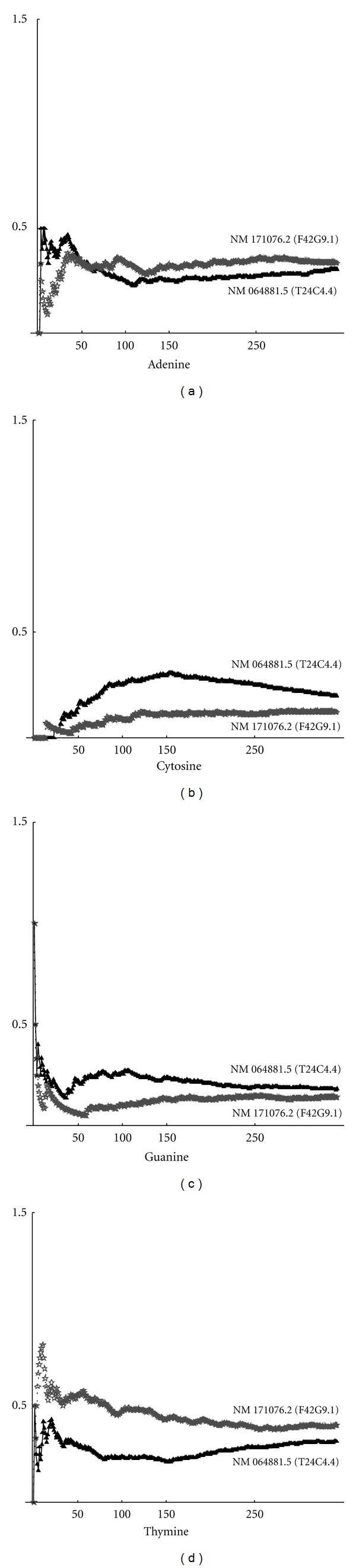
Max-min frequency curves for noncoding sequences.

**Figure 4:**
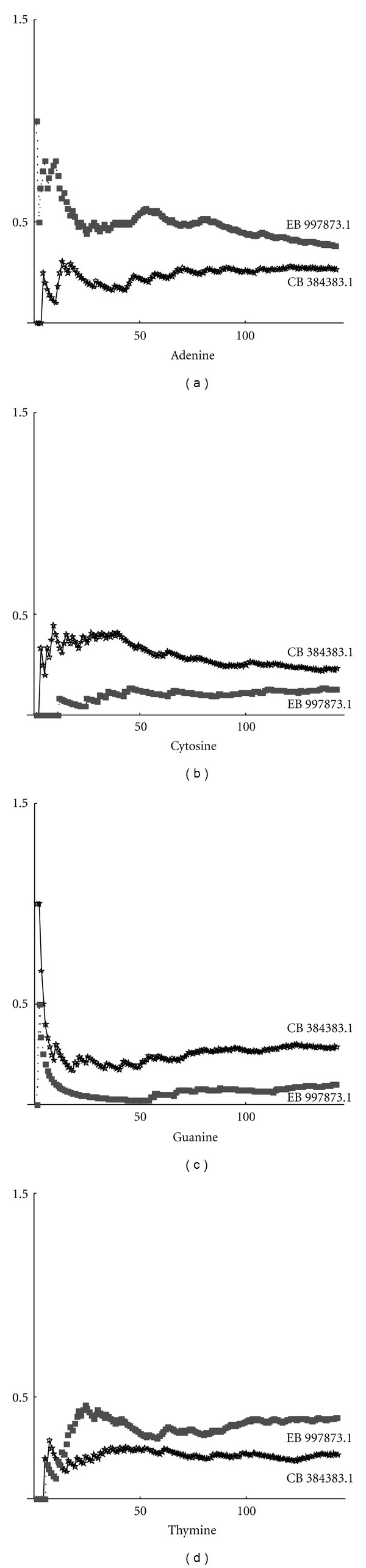
Max-min frequency curves for coding sequence.

**Figure 5:**
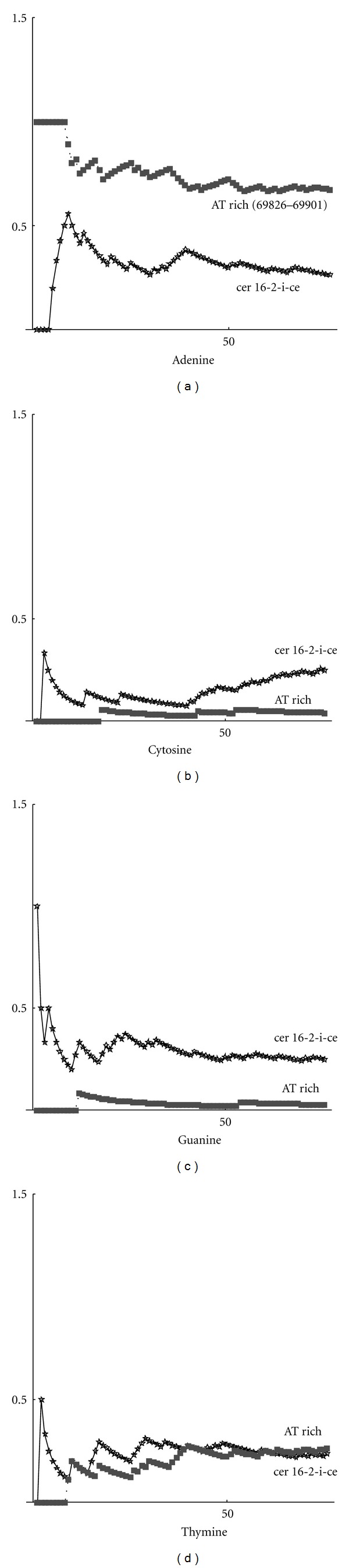
Max-min frequency curves for repeats sequence.

**Figure 6:**
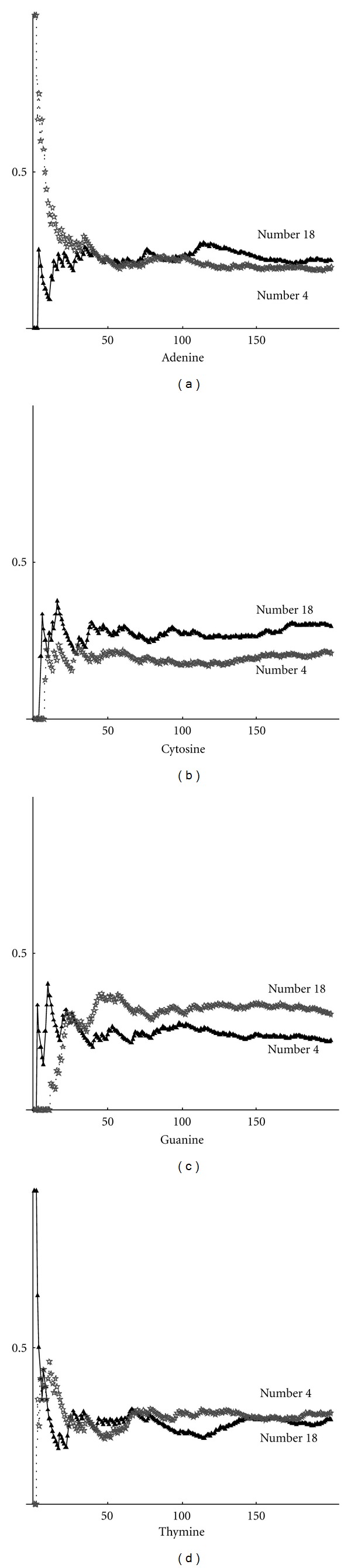
Max-min frequency curves for random sequences.

 We investigated the complexity of the nucleotide sequences. In all cases, we obtained that the curve of higher complexity corresponds to the sequence with the highest fractal dimension. Thus, we can draw the conclusion that complexity and fractal dimension are equivalent parameters for studying the complexity. These results depend on the distribution of nucleotides. By using the definition (6), we can compute the frequency distribution on a sequence. Below are shown the frequencies for each nucleotide (adenine, cytosine, guanine, thymine). In particular, in Figure 2, the max-min curves for frequencies on the whole gene sequence are plotted. It can be seen that, in this case, adenine and cytosine tend to have the same value, while thymine and guanine maintain a significant distance between the max and min curves. Max-min frequency curves for noncoding sequences are shown in Figure 3. By taking into account the values of fractal dimensions, as given in Tables 1 and 2, we can observe that the higher frequency of cytosine corresponds to the higher fractal dimension. Thymine, instead, is more present in sequences with low fractal dimension. In Figure 4, the curves for max-min frequency of coding sequences are drawn. It can be seen, also in this case, that adenine and thymine are more present in the sequence with lower fractal dimension. As before, cytosine is more present in sequences with higher fractal dimension. Repeats and random sequences are given in Figures 5 and 6, respectively. In the first case for adenine, we have more frequencies rate for the low fractal sequence, while for cytosine we have more frequencies rate for the high fractal sequence. For random sequences, we have that the cytosine is more frequent in the sequence that has the highest value of fractal. 

**Figure 7:**
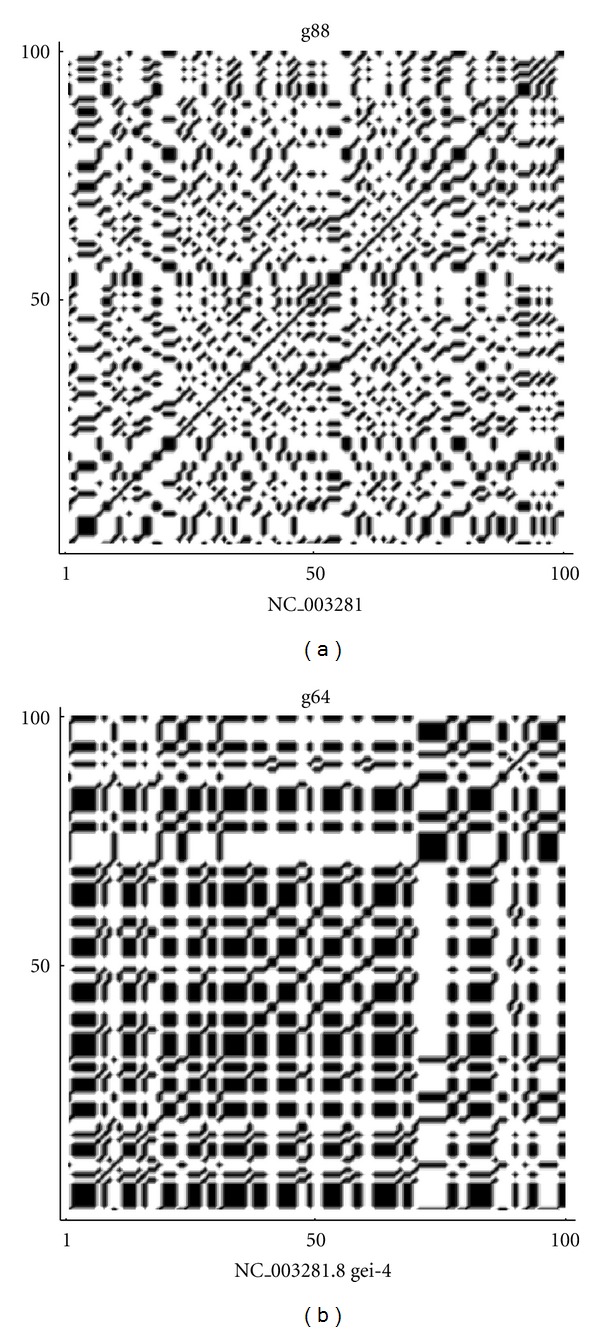
Autocorrelation plots on the whole sequence gene corresponding to max and min values of fractal dimension in (a) and (b), respectively.

**Figure 8:**
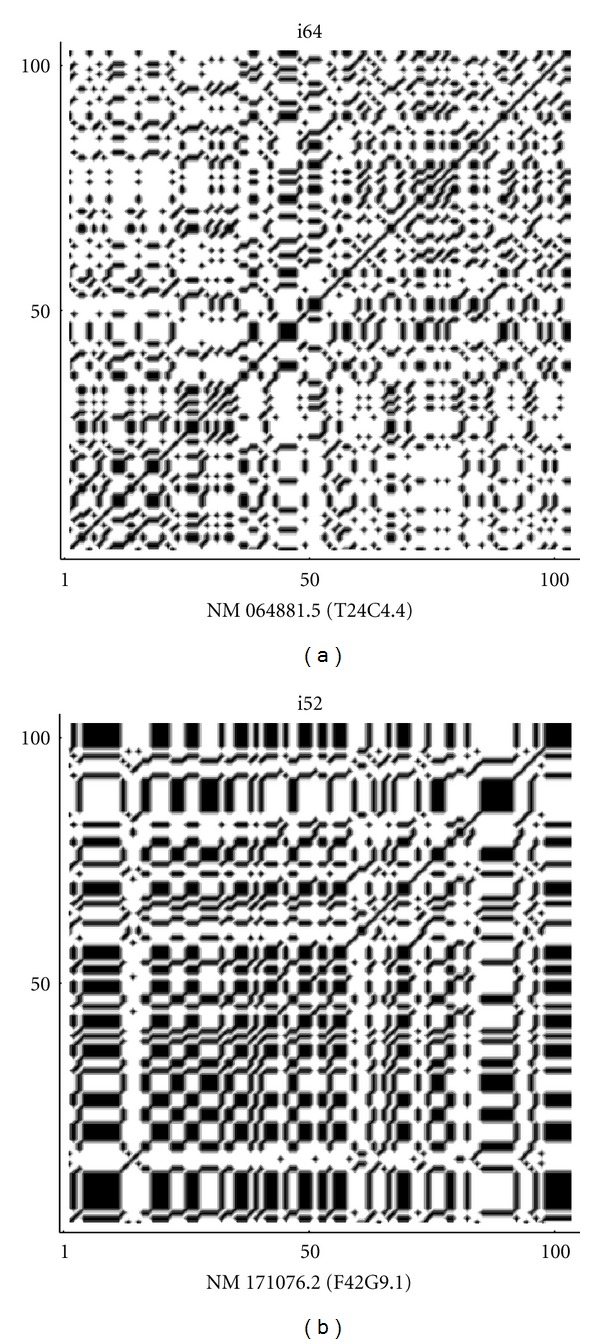
Autocorrelation plots on the noncoding sequences corresponding to max and min values of fractal dimension in (a) and (b), respectively.

**Figure 9:**
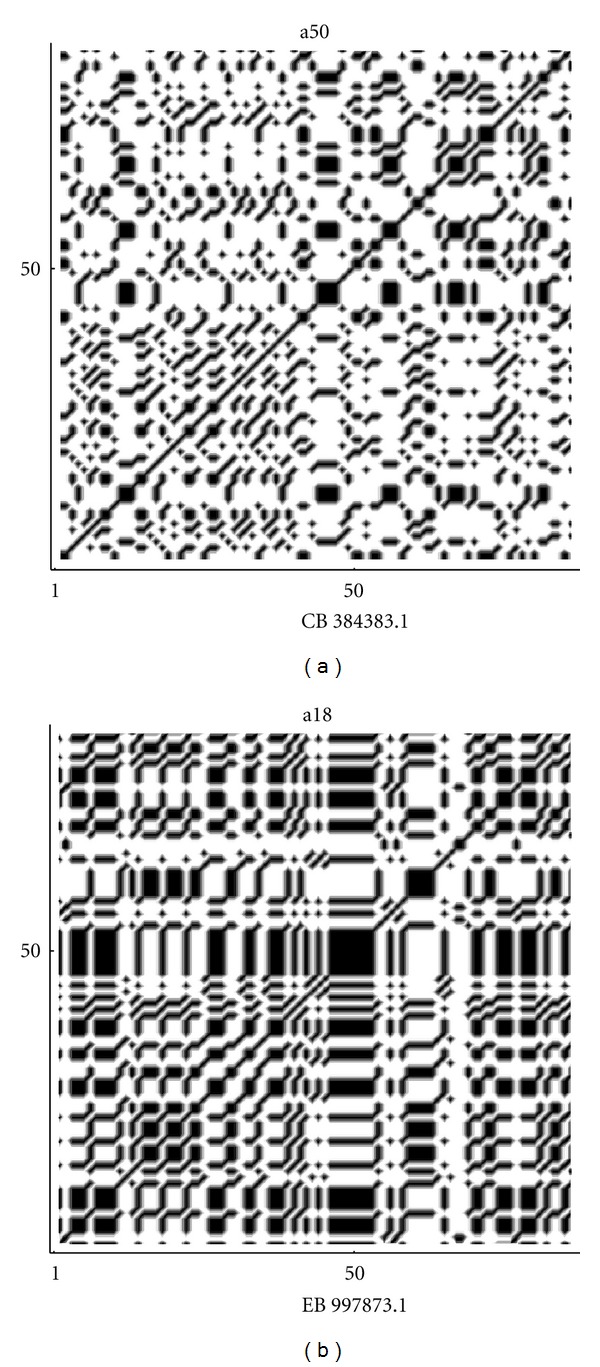
Autocorrelation plots on the coding sequences corresponding to max and min values of fractal dimension in (a) and (b), respectively.

**Figure 10:**
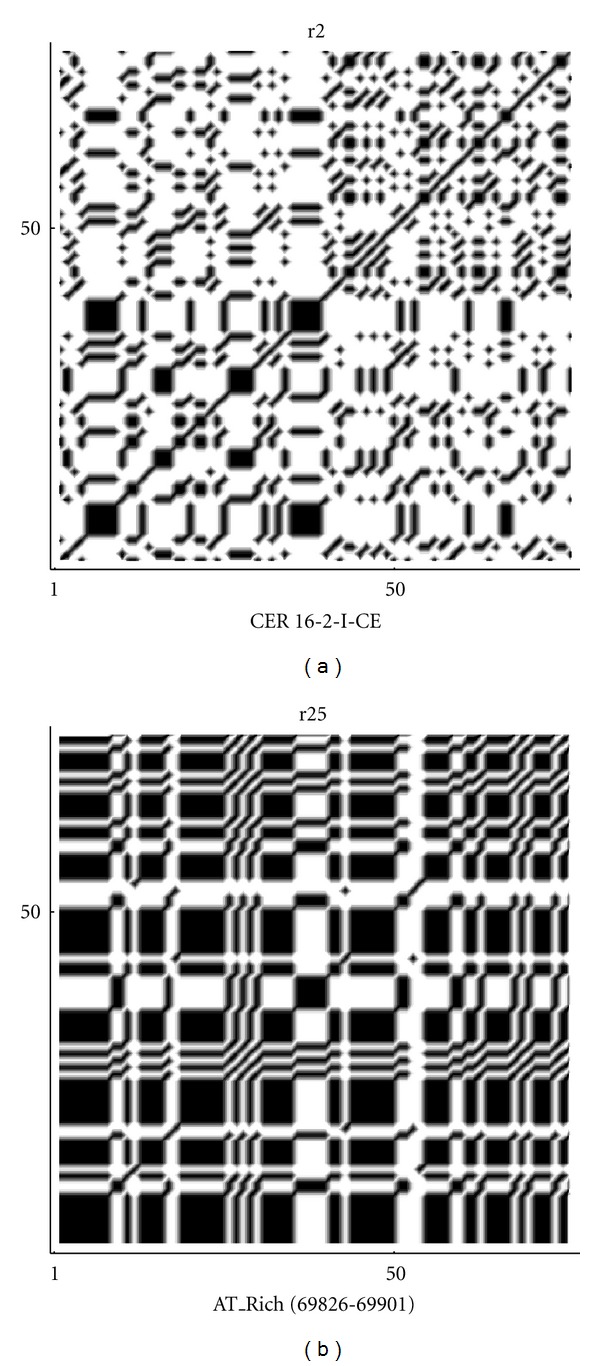
Autocorrelation plots on the repeats sequences corresponding to max and min values of fractal dimension in (a) and (b) respectively.

**Figure 11:**
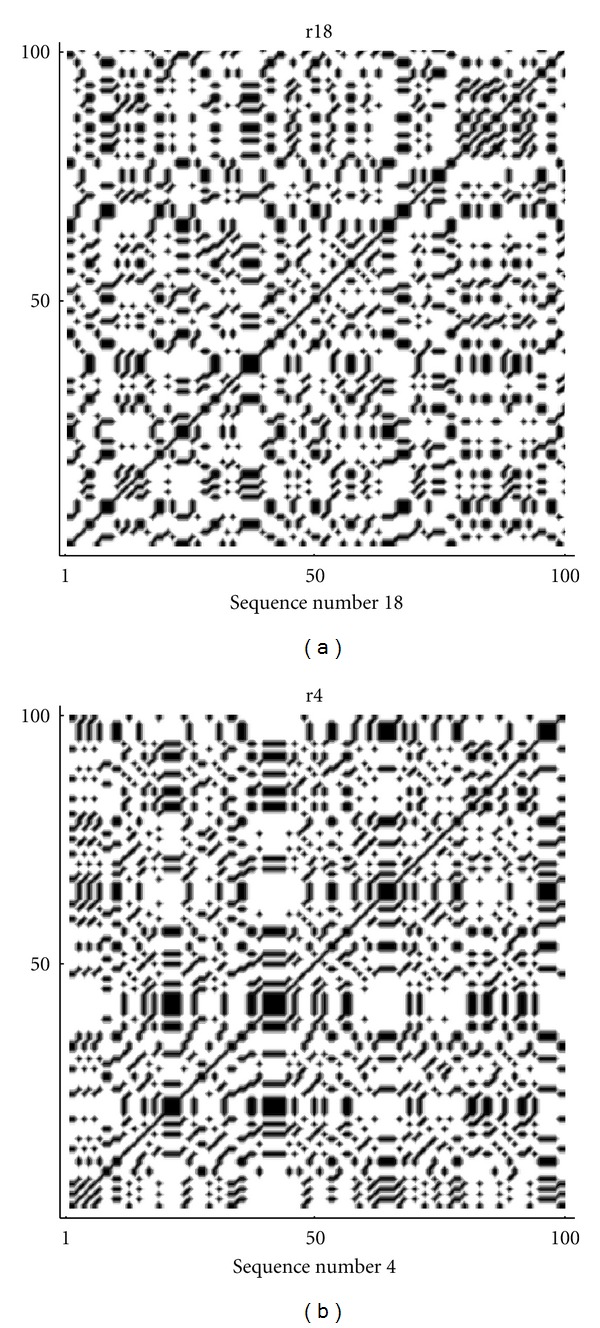
Autocorrelation plots on the random sequences corresponding to max and min values of fractal dimension in (a) and (b), respectively.

 By the frequency analysis and the results of Tables 1 and 2 on the fractal dimension we can see that there is a correspondence between the frequencies of nucleotides and the fractal dimension. So that, sequences that show a lower fractal dimension have always a higher frequency for the adenine and thymine (in most cases), while the cytosine is more frequent in high fractal sequences. Almost the same results are true also for random sequences, especially for the thymine and cytosine. According to (5), the indicator map of the *N*-length sequence can be easily represented by the *N* × *N* sparse matrix of binary values {0, 1} and this matrix can be visualized by the following (autocorrelation) dot-plots [20, 22] of Figures 7, 8, 9, 10, and 11.  Figure 11(a) shows the sequences (of Table 1) with max value of fractal dimension, while in Figure 11(a), there are the sequences of Table 2 with min value of fractal dimension. We can see that also in these plots the distribution of nucleotides gives rise to some typical patterns.

All sequences with low fractal dimension (Figure 11(b)) turn out to have an important presence of nucleotide correlation, this feature is less present in the sequences with higher fractal dimension, where we expect to have a more complex structure of the sequence.

## 4. Discussion

In this work, by means of statistical parameters such as indicator matrix, complexity, frequency, and fractal dimension, the different types of sequences (repeats, coding, noncoding, whole gene, random) of chromosome 3 (the one with the highest fractality) of the *C*. *elegans* have been analyzed. Our attempt was to give a statistical classification of these sequences and to understand the complexity of the sequences as a function of the nucleotides' distribution. By using (7) the values of the fractal dimension for all sequences are obtained. In detail, it was observed that the repeats sequences (which do not code for proteins) have a higher variability of values, since they assume the minimum and maximum on all sequences in the *C*. *elegans*. This leads us to analyze the role and the functional meaning of the repeats within the sequences of genes. Thereafter, we have verified the equivalence, with respect to the complexity, between the fractal dimension and complexity, since the sequences with highest fractality appear to have also a greater degree of complexity. Through the frequency distribution of nucleotide, it was noticed that the adenine is more present in sequences having a lower fractal dimension and, in particular, for the one being in absolute the lowest fractal (AT RICH). This result seems to be dependent on the fact that the sequence is made up of only 2 nucleotides, that is, adenine and thymine. Cytosine, instead, appears to be the most frequent nucleotide in the sequence with the highest fractal value and in particular for the sequence CER 16-2-i-CE. These results lead us to conjecture that there is a correlation between fractal dimension and the frequency of nucleotides such as adenine and cytosine. The information contents of a sequence of nucleotides depend on the different distribution of nucleotides, so that two sequences having the same nucleotides which are distributed according to two different permutations might have two different complexities (fractal dimension). In future work, this aspect of the different organization within the sequence will be further analyzed. Moreover, these results must be confirmed in other organisms which are evolutionarily distant from each other to better investigate the findings so far. At the moment, the obtained results were compared with some random sequences, which have a nucleotide random distribution, and in that case, we have obtained a significant correspondence with the complexity of the nucleotide sequences.

## Figures and Tables

**Table 1:** Max value of fractal dimension of sequences.

Type of sequence	Max value of fractal dimension	Tag of genomic sequence
Whole sequence of gene	1.29850	NC 003281 (T19C3.7)
Noncoding	1.29808	NM 064881.5 (T24C4.4)
Coding	1.30639	CB 384383.1
Repeats	1.31280	CER 16-2-i-CE
Random sequence	1.28452	Number 18

**Table 2:** Min value of fractal dimension of sequences.

Type of sequence	Min value of fractal dimension	Tag of genomic sequence
Whole sequence of gene	1.27016	NC 003281.8 gei-4
Noncoding	1.27494	NM 171076.2 (F42G9.1)
Coding	1.27846	EB 997873.1
Repeats	1.24155	AT rich (69826–69901)
Random sequence	1.28201	Number 4
